# Small-Molecule-Directed Endogenous Regeneration of Visual Function in a Mammalian Retinal Degeneration Model

**DOI:** 10.3390/ijms25031521

**Published:** 2024-01-26

**Authors:** Daphna Mokady, Jason Charish, Patrick Barretto-Burns, Kenneth N. Grisé, Brenda L. K. Coles, Susanne Raab, Arturo Ortin-Martinez, Alex Müller, Bernhard Fasching, Payal Jain, Micha Drukker, Derek van der Kooy, Matthias Steger

**Affiliations:** 1 Endogena Therapeutics, Inc., 661 University Ave, Toronto, ON M5G 0B7, Canadapat.barretto.burns@gmail.com (P.B.-B.);; 2 Department of Molecular Genetics, University of Toronto, Donnelly Centre Rm 1110, 160 College Street, Toronto, ON M5S 3E1, Canada; 3 Endogena Therapeutics, AG, Binzmuehlestrasse 170 d, CH-8050 Zuerich, Switzerland

**Keywords:** small molecule, regenerative medicine, inherited retinal diseases, retina, photoreceptors, functional recovery

## Abstract

Degenerative retinal diseases associated with photoreceptor loss are a leading cause of visual impairment worldwide, with limited treatment options. Phenotypic profiling coupled with medicinal chemistry were used to develop a small molecule with proliferative effects on retinal stem/progenitor cells, as assessed in vitro in a neurosphere assay and in vivo by measuring Msx1-positive ciliary body cell proliferation. The compound was identified as having kinase inhibitory activity and was subjected to cellular pathway analysis in non-retinal human primary cell systems. When tested in a disease-relevant murine model of adult retinal degeneration (MNU-induced retinal degeneration), we observed that four repeat intravitreal injections of the compound improved the thickness of the outer nuclear layer along with the regeneration of the visual function, as measured with ERG, visual acuity, and contrast sensitivity tests. This serves as a proof of concept for the use of a small molecule to promote endogenous regeneration in the eye.

## 1. Introduction

Among degenerative retinal diseases, inherited retinal rod–cone dystrophies, including retinitis pigmentosa (RP), consist of a clinically and genetically heterogeneous group of irreversible retinopathies characterized by slow and progressive death of photoreceptors, with initial rod loss, followed by degeneration of cones [1]. Given the genetic and mechanistic heterogeneity underlying retinal degeneration, therapy-driven recovery of photoreceptors may be a direct and broadly applicable means of slowing disease progression and improving visual function.

Clinically, once photoreceptor function is lost, the capacity for restoration is limited. Recent advances in gene therapy have demonstrated that improving visual function is possible [2,3]; however, due to the genetic heterogeneity of inherited retinal diseases (IRDs), this treatment option will be inaccessible to most patients for the near future. Other developing strategies that exploit stem cell advances, such as cell-based transplantations, while promising, face additional challenges (proper selection of cell source and type for transplantation, difficulty in achieving integration of transplanted cells, possible immune responses, and severe adverse events) on their path to the clinic [4]. An alternative approach, which is gaining traction as a potential option in disorders impacting various tissues [5,6], is the use of small molecules to promote the endogenous regeneration of lost tissue function.

One way to gauge the potential of a small molecule to promote functional regeneration in vivo in the adult mammalian retina is to investigate its effects on a population of cells that, at least in some lower vertebrates, can participate in retinal regeneration during adulthood [7]. Retinal stem/progenitor cells (RSPCs) are multipotent central nervous system precursors that give rise to the retina during development, though their continued presence in vivo in adult mammalian eyes remains debated [8,9,10]. One possible source of adult mammalian RSPCs is a population of cells residing in the ciliary epithelium of the adult eye [9,11,12,13]. When single ciliary epithelium cells are isolated from adult mammalian eyes and cultured in vitro, they form large, free-floating neurospheres capable of both self-renewal and subsequent directed differentiation into neural retinal cell types [11,12,13].

Our aims were, therefore, to (i) utilize in vitro and in vivo assays to demonstrate that a small molecule can promote proliferation of this subpopulation of ciliary body cells and (ii) determinate if local administration of the small molecule in an in vivo mammalian model of retinal degeneration can promote functional regeneration. Together, this may serve as a proof of concept for the therapeutic use of a small molecule to promote controlled endogenous functional recovery in the degenerated retina.

## 2. Results

### 2.1. CompoundA Promotes In Vitro and In Vivo RSPC Proliferation

**Figure 1:**
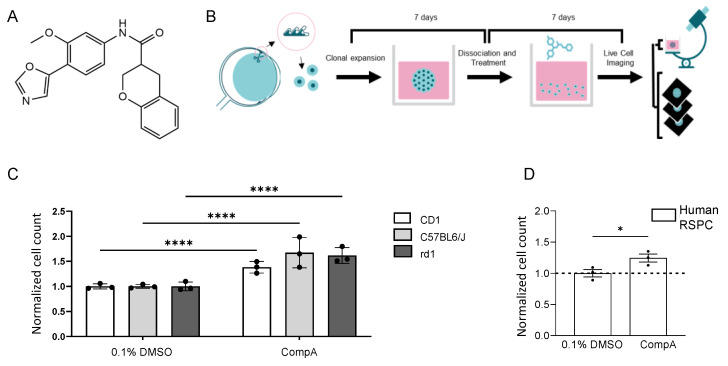
RSPC proliferation can be increased in vitro using a small molecule. (**A**) Molecular structure of CompA. (**B**) Schematic representation of phenotypic assay. Primary neurospheres derived from mouse ciliary epithelium were grown for 7 days. Primary spheres were then dissociated and cultured as a single-cell suspension in the presence of compounds for 7 days. The phenotypic readout is the number of objects in the cultures of secondary neurospheres. (**C**) CompA (5 μM) increases the proliferation of mouse RSPCs in both albino (CD1),pigmented (C57BL/6J) and rd1 8–10-week-old mice compared with DMSO controls. *n* = 3 for each; **** *p* < 0.0001; two-way ANOVA with corrections for multiple comparisons. Values are normalized to the mean of 0.1% DMSO which is set as 1. (**D**) Exposure of primary human RSPCs to CompA (5 μM) increases proliferation. *n* = 3 for each; * *p* < 0.05; *t*-test. All results presented as means ± SEMs. Values are normalized to the mean of 0.1% DMSO which is set as 1.

Signaling pathways such as TGFβ, Wnt, FGF, mTor, and Hippo are major drivers of neuroectoderm development [14,15,16,17], and cell-permeable small-molecule-based manipulation of these pathways can influence neural stem/progenitor cell proliferation, differentiation, and/or survival [18,19,20]. Molecular information from such compounds was used with machine learning and computer-aided drug design [21,22,23], together with standard medicinal chemistry techniques and considerations for drug likeness and molecular diversity, for the identification of CompoundA (CompA). CompA is a cell-permeable small molecule with drug-like physicochemical properties (MW: 350.37; clogP: 3.7; PSA: 74) enabling in vitro, as well as in vivo, investigation of its effect on RSPCs. Given that the chemical structure of CompA (Figure 1A) may have kinase inhibitory activity, a kinase panel (KinaseProfiler™; Eurofins Discovery services, Celle-Lévescault, France) was performed to identify possible molecular targets. The following kinases were inhibited by more than 75% with 5 µM CompA: PKG1α, ROCK-I, p70S6K, and PKA (Supplementary Figure S1A). In addition to the roles in the aforementioned signaling pathways, these kinases have been implicated in retinal cell proliferation, function, and pathophysiology [24,25,26,27].

The in vitro phenotypic assay involved the harvesting and culturing of the ciliary body from adult mice to generate primary neurospheres (Figure 1B). After seven days in culture, the resulting neurospheres were collected and dissociated enzymatically and mechanically to form a stem/progenitor single-cell suspension, which was then cultured for seven days with the test item or control (secondary neurospheres). Increased proliferation was indicated by significantly increased object counts in the secondary neurosphere cultures [28,29]. In the described assay, after incubation with CompA, object counts were significantly increased compared with controls (Figure 1C), without affecting nucleus size (Supplementary Figure S1B), whereas the vehicle control (DMSO) did not affect proliferation (Supplementary Figure S1C). CompA also significantly increased the number of tertiary spheres and tertiary object counts (Supplementary Figure S1D,E). Comparable increases in object counts were seen in the secondary neurosphere cultures derived from various genetic backgrounds, including mice homozygous for the severe retinal degeneration-causing Pde6brd1 mutation (rd1 mice; Figure 1C). CompA also significantly increased object counts when cells were either derived from aged mice (Supplementary Figure S1F) or derived from adult human eyes (Figure 1D), indicating that the proliferative effect may be translatable from murine to human cells. Previous exposure of secondary neurospheres to CompA did not affect the ability of the cells to express a cone marker and cone-signature genes when cells were subsequently subjected to in vitro conditions conducive to differentiation into cone-like cells [30] (Supplementary Figure S1G). Finally, using rd1-derived neurospheres, the EC50 for CompA was determined to be ~0.5 μM (Supplementary Figure S1H). Altogether, CompA promoted RSPC proliferation without interfering with subsequent differentiation, with a potency suitable for intravitreal (IVT) administration.

To gain insight into the cellular pathways targeted by CompA, additional phenotypic profiling was performed in twelve non-retinal human primary cell systems (BioMAP^®^ Diversity PLUS^®^; Eurofins Discovery Services; Supplementary Table S1), which identified various CompA-mediated immunomodulatory and tissue-remodeling/anti-fibrotic-related activities, as well as antiproliferative effects on coronary artery smooth muscle cells, endothelial cells, and fibroblasts, indicating that CompA does not indiscriminately promote proliferation (Supplementary Figure S2A,B). Tissue-remodeling activities included modulation of tissue inhibitor of metalloproteinase (TIMP)-1 and TIMP-2 in the wound healing and inflammation system (HDF3CGF), as well as decreased expression of urokinase plasminogen activator surface receptor (uPAR) in the vascular inflammation system (3C). An unsupervised search for similar compound profiles in a BioMAP reference database (>4500 compounds) did not generate any matches that reached the threshold for relevant similarity (Pearson correlation coefficient ≥ 0.7 [31]; Supplementary Table S2), suggesting mechanistic distinctiveness and supporting the use of a phenotypic approach. Nonetheless, the closest matches with Pearson correlation coefficient <0.7 included an inhibitor of mTor subtypes mTORC1 and mTORC2 (Torin-1), as well as several ROCK inhibitors (H1152 and Hydroxyfasudil), which is in line with the identification of the kinases ROCK1 and p70S6K as targets of CompA (Supplementary Figure S1A).

Prior to investigating the effects of CompA on the regeneration of retinal and visual function, we first sought to (i) investigate the effects of CompA on in vivo ciliary body proliferation and (ii) examine the effects of CompA in a relevant disease context. During murine eye development, there is a subpopulation of multipotent Msx1-expressing ciliary marginal zone cells that produce multiple neural retinal progenies, including photoreceptors [32]. Postnatally, a subpopulation of ciliary body cells maintains Msx1 expression. While these postnatal cells are not thought to participate in retinogenesis [32], they do represent part of the ciliary body-derived population of cells capable of forming secondary neurospheres in vitro [11]. Using an inducible fluorescent Msx1CreERT2;tdTomato mouse line, tamoxifen induction was performed on postnatal days (P)11-P14 to label cells expressing Msx1 at these time points with tdTomato, allowing for the in vivo quantification of labelled postnatal Msx1-lineage cells during adulthood.

**Figure 2:**
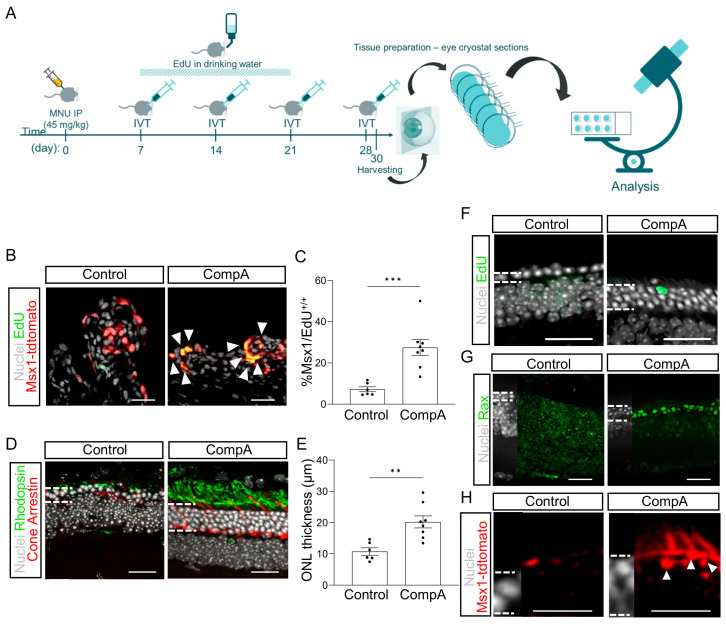
RSPC proliferation can be increased in vivo using a small molecule. (**A**) Schematic representation of experimental design. Msx1Cre^ERT2^; tdTomato mice were given 45 mg/kg MNU (IP) to induce retinal degeneration. Four weekly intravitreal (IVT) injections of vehicle control (BSS) or CompA were administered. Thirty days after MNU, eyes were harvested and cryosectioned. (**B**) Representative images of Msx1^+^ (red) and EdU^+^ (green) cells in the ciliary body. White arrows indicate nuclei double-labelled for Msx1-tdTomato and EdU. (**C**) Quantification of (**B**) *n* = 6 for vehicle control and *n* = 8 for CompA; *** *p* < 0.001; Student’s *t*-tests with Welch’s correction. (**D**) Representative images from the mid-periphery (900 µM from optic nerve head) of a vehicle control-treated and CompA-treated retina stained for rhodopsin (green), cone arrestin (red), and nuclei (grey). (**E**) CompA treatment increased ONL thickness in mice with MNU-induced retinal degeneration. *n* = 6 for vehicle control and *n* = 8 for CompA; ** *p* < 0.01; Student’s *t*-tests with Welch’s correction. (**F**) EdU^+^ cells can be detected in the ONL of CompA-treated eyes (image approximately 600 µM from optic nerve head). (**G**) Treatment with CompA increases the number of cells within the outer nuclear layer (ONL) of the neural tip (periphery) that are positive for a retinal progenitor marker (Rax; green). (**H**) Msx1-tdTomato^+^ cells (red) with processes consistent with inner segments could be found in the mid-periphery of CompA-treated retinas (image approximately 1100 uM from optic nerve head) but not vehicle controls. Dashed lines indicate border of ONL. Some Msx1-tdTomato signal could be seen in the outer limiting membrane of both vehicle control and CompA retinas. White dashed lines (G-M) indicate the approximate border of the ONL. Scale bars = 50 μm. All results presented as means ± SEMs.

To mimic a relevant disease context, we utilized N-methyl-N-nitrosourea (MNU; 45 mg/kg) to induce photoreceptor death, a widely used preclinical animal model of retinal degeneration [33,34]. A single intraperitoneal (IP) injection of MNU causes selective photoreceptor death, which manifests as a rapid decrease in outer nuclear layer (ONL) thickness primarily taking place over one week [35,36]. Photoreceptor degeneration was allowed to proceed for seven days, after which mice were given four weekly bilateral IVT injections of either CompA or vehicle control (Figure 2A). During the treatment weeks, 5-ethynyl-2′-deoxyuridine (EdU) was provided ad libitum via drinking water to label proliferating cells. A change in the proportion of Msx1-tdTomato+ cells that were also positive for EdU within the ciliary epithelium was used as a readout for whether CompA influenced the proliferation of this cell subpopulation.

Two days after the fourth IVT injection, we observed a significant increase in the proportion of Msx1-lineage cells that were EdU+ in CompA-treated eyes compared to the vehicle controls (Figure 2B,C) without affecting the total cell count within the ciliary body (Supplementary Figure S3B). Histological analysis indicated that the thickness of the ONL was significantly greater in CompA-treated eyes compared to the vehicle controls (Figure 2D,E), coupled with improved photoreceptor morphology (markers of rod or cone photoreceptors; Figure 2D), increased cone numbers (Figure 2D and Figure S3C), and conserved outer plexiform layer morphology (Supplementary Figure S3D). Although it remains unclear how the increased proliferation of Msx1+ cells related to the improved ONL thickness observed following CompA treatment, we did observe occasional EdU+ cells within the ONL of CompA-treated retina but not vehicle control-treated retina (Figure 2F). This limited number of EdU+ cells suggests either incomplete EdU labelling or that the increased ONL thickness is not predominantly due to new cells. Otx2 is an early differentiation marker that is expressed in the nucleus of retinal progenitor cells and promotes photoreceptor differentiation [37,38,39]. CompA treatment led to a qualitative increase in the number of cells within the ONL of the neural tip that were positive for nuclear staining with Otx2 [40] (Supplementary Figure S3E). Rax is a transcription factor necessary for normal retinal development and is highly expressed in vivo in murine retinal progenitor cells and maturing photoreceptors, with photoreceptor expression becoming reduced in adulthood [41]. We observed strong Rax expression, relative to vehicle controls, in a subset of cells within the ONL of the neural tip in CompA-treated eyes only (Figure 2G). These findings are consistent with the interpretation that CompA increases retinal progenitor cell levels within the ONL. Integrated Msx1-lineage cells could also be found in the outermost layer of the ONL, with processes being consistent with inner segment morphologies, in CompA-treated retina only (Figure 2H). Together, this demonstrates that CompA produces an in vivo effect that is comparable to those observed in the in vitro assay (Msx1-lineage cell proliferation). We hypothesized that these effects may be sufficient to recover visual function.

### 2.2. Following Retinal Degeneration CompoundA Treatment Restores Retinal Function

**Figure 3:**
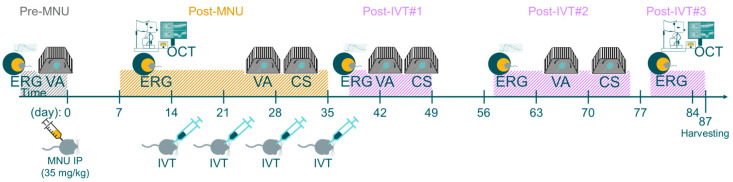
Schematic of functional visual tests. Schematic representation of experimental timeline. Adult C57BL/6J mice were given 35 mg/kg MNU IP to induce retinal degeneration. Two weeks after MNU, mice were given four weekly intravitreal (IVT) injections of CompA (0.14 µg per injection) or vehicle control. Contrast sensitivity (CS) and visual acuity (VA) were assessed prior to MNU administration (pre-MNU; VA only), prior to completion of treatment (post-MNU) and at various time points following completion of IVT treatment (post-IVT#1 and post-IVT #2). OCT imaging was performed 14 and 87 days post-MNU. Photopic and scotopic flash-ERG recordings were performed prior to MNU (pre-MNU), following MNU (post-MNU), and at various time points following treatment completion (post-IVT #1–3).

To assess the impact of four weekly bilateral IVT injections of either vehicle control or CompA on the visual function of mice with retinal degeneration, three separate tests were used: (i) contrast sensitivity, which, when assessed under conditions like those in the present study, primarily depends on cone-driven visual function [42]; (ii) visual acuity (cone-driven); and (iii) flash-ERG recordings, which can detect both rod- and cone-driven impairment. To achieve this, we undertook a study using adult C57BL/6J mice with MNU-induced retinal degeneration (Figure 3). Here, retinal degeneration was induced using an optimized dosage of 35 mg/kg MNU, which causes severe retinal degeneration but also preserves sufficient ONL to make detection of residual visual function more likely. Although severe retinal degeneration was largely complete by day seven (Supplementary Figure S3A), we allowed retinal degeneration to progress unperturbed for an additional week (two weeks total) to ensure that primary MNU-induced photoreceptor death was largely complete prior to treatment initiation [36]. At fourteen days following MNU, all mice displayed severe bilateral retinal degeneration with no significant difference in central ONL thickness between the CompA and vehicle control groups as assessed using optical coherence tomography (OCT) imaging (Supplementary Figure S3A). At this time point, four weekly bilateral IVTs of either vehicle control of CompA were administered.

**Figure 4:**
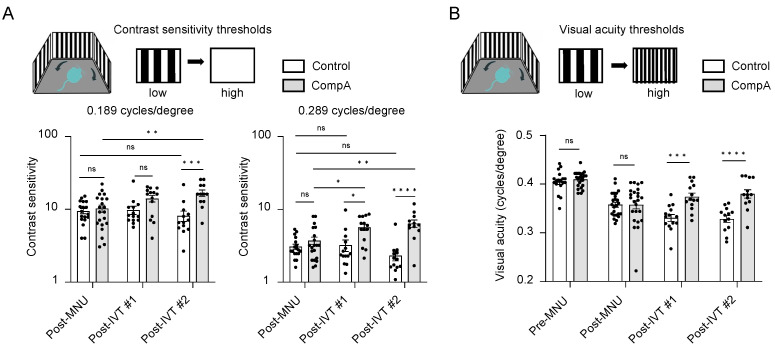
CompA treatment restores contrast sensitivity (CV) and visual acuity (VA) following retinal degeneration. (**A**) Prior to treatment completion (post-MNU), there were no significant differences in contrast sensitivity between vehicle control and CompA eyes (*n* = 18 eyes from 10 animals and 20 eyes from 11 animals, respectively) at 0.189 cycles/degree (left) or 0.289 cycles/degree (right). Following treatment, compared to vehicle controls, CompA eyes had significantly improved CS at post-IVT #1 (0.289 cycles/degree only; *n* = 14 eyes for both (from 10 animals for vehicle control and from 9 animals for CompA; * *p* < 0.05) and at post-IVT #2 (*n* = 13 eyes from 9 animals for vehicle controls and *n* = 12 eyes from 8 animals for CompA; *** *p* < 0.001; **** *p* < 0.0001; *ns* = non-significant). Only CompA eyes had improved CS over time when comparing post-MNU to post-IVT time points (* *p* < 0.05; ** *p* < 0.01). Significance determined by two-way ANOVA followed by Sidak’s multiple comparison test. (**B**) There were no significant differences in VA between vehicle control and CompA eyes pre-MNU (*n* = 18 eyes from 9 animals and 24 eyes from 12 animals, respectively) and post-MNU (*n* = 24 eyes from 12 animals for both). VA was significantly improved in CompA-treated eyes compared to vehicle-treated control eyes at post-IVT #1 (*n* = 14 for both vehicle controls and CompA; from 10 animals for control and from 9 animals for CompA; *** *p* < 0.001) and at post-IVT #2 (*n* = 13 for vehicle control and CompA, from 9 and 8 animals, respectively; **** *p* < 0.0001; *ns* = non-significant). Significance determined by two-way ANOVA followed by Sidak’s multiple comparison test. All results are presented as individual data points together with the mean ± SEM. All results presented as mean ± SEM.

Contrast sensitivity (CS) measures the ability to distinguish between fine increments in light versus dark and depends on the processing of photoreceptor-driven signals. To assess CS in mice, we measured optomotor tracking response thresholds using the OptoMotry (CerebralMechanics) system. Contrast thresholds were measured at two spatial frequencies which approximate (i) the maximum spatial frequency at which all mice still elicited recordable optomotor responses at maximum contrast following MNU degeneration (0.289 cycles/degree) and (ii) the spatial frequency at which wild type C57BL/6J mice normally display the highest contrast sensitivity (0.189 cycles/degree [43]). Following MNU-induced retinal degeneration, but prior to completion of IVT treatment (post-MNU; Figure 3 and Figure 4A), there was no significant difference in the CS of the CompA group compared with the vehicle control group at either 0.189 or 0.289 cycles/degree, indicating that both groups were equally impaired. Following the completion of IVT treatment (post-IVT #1; Figure 3 and Figure 4A), there was a significant improvement in CS in CompA-treated eyes compared to the vehicle controls at 0.289 cycles/degree. At a later time point (post-IVT #2), there was a significant improvement in CS at both 0.189 and 0.289 cycles/degree in CompA-treated eyes compared to the vehicle controls. Furthermore, at both spatial frequencies, there was a significant improvement in CompA-treated eyes between the post-MNU and post-IVT time points, indicating that CS was improving over time following treatment in this group only.

Visual acuity (VA) was also assessed in mice by measuring optomotor response thresholds in the OptoMotry system [44]. Prior to MNU-induced retinal degeneration (pre-MNU; Figure 3 and Figure 4B), there were no significant differences in VA between the two groups. Following MNU-induced retinal degeneration but prior to completion of IVT treatment (post-MNU; Figure 3 and Figure 4B), there were also no significant differences in the VA of the CompA group compared with the vehicle control group, indicating that both groups had equally impaired VA in response to MNU. Following IVT treatment (post-IVT #1 and post-IVT #2 time points; Figure 3 and Figure 4B), there was a significant improvement in VA in CompA-treated eyes compared with vehicle control-treated eyes.

**Figure 5:**
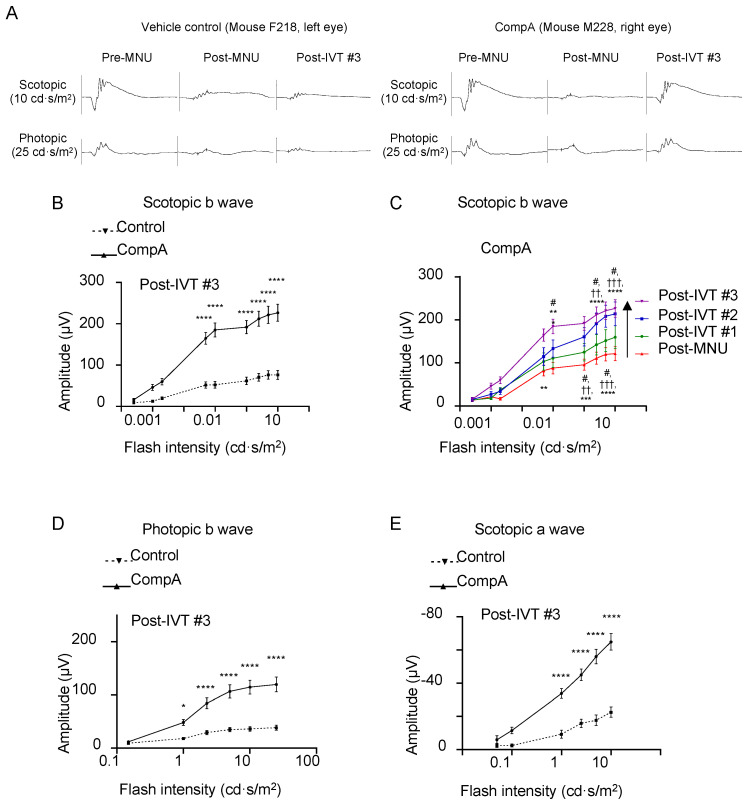
CompA treatment promotes regeneration of electroretinogram (ERG) responses following retinal degeneration. (**A**) Representative scotopic (**top**) and photopic (**bottom**) ERG traces of a single vehicle control eye (**left**) and a single CompA eye (**right**) at pre-MNU, post-MNU, and post-IVT#3. (**B**) At post-IVT#3, scotopic b wave amplitudes were significantly increased in CompA eyes compared to vehicle controls (*n* = 13 eyes from 9 animals for vehicle control and *n* = 12 eyes from 8 animals for CompA; **** *p* < 0.0001; two-way ANOVA followed by Sidak’s multiple comparison test). (**C**) Over time, there was an improvement in scotopic b wave amplitudes in CompA-treated eyes between post-MNU and post-IVT #3 (*n* = 22 and 13, respectively; * = post-MNU vs. post-IVT #3; † = post-MNU vs. post-IVT #3; # = post-IVT #1 vs. post-IVT #3; ** *p* < 0.01, *** *p* < 0.001, **** *p* < 0.0001, # *p* < 0.05, †† *p* < 0.01, ††† *p* < 0.001; two-way ANOVA followed by Sidak’s multiple comparison test). (**D**) At post-IVT#3, photopic b wave amplitudes were significantly increased in CompA eyes compared to vehicle controls (* *p* < 0.05; **** *p* < 0.0001; two-way ANOVA followed by Sidak’s multiple comparison test). (**E**) At post-IVT#3, a-wave amplitudes were significantly increased in CompA eyes compared to vehicle controls (**** *p* < 0.0001; two-way ANOVA followed by Sidak’s multiple comparison test). All results are presented as mean ± SEM.

To measure the light-evoked, photoreceptor-driven electrical activity of the eye, flash-ERG recordings were performed prior to MNU administration (pre-MNU), following MNU but prior to treatment (post-MNU; approx. 1.5 weeks MNU), and at various time points following the completion of IVT treatment (post-IVT #1 at approx. 5.5 weeks following MNU, post-IVT#2 at approx. 8.5 weeks following MNU, and post-IVT#3 at approx. 11.5 weeks following MNU; Figure 3). Representative photopic and scotopic traces of an individual vehicle control eye and a CompA eye illustrate how CompA treatment restored flash-ERG responses recorded under both scotopic and photopic conditions (Figure 5A and Figure S4A,B). In flash-ERG waves, b-waves reflect downstream photoreceptor-driven changes in the activity of bipolar interneuron retinal cells. Prior to MNU-induced retinal degeneration, both vehicle control and CompA groups had similar b-wave amplitudes for all flash intensities recorded under scotopic conditions, whereas post-MNU (and prior to IVT treatment), both groups had equally impaired amplitudes following MNU-induced retinal degeneration (Supplementary Figure S5A). Following four weekly IVT injections, CompA-treated eyes had significantly improved b-wave amplitudes at all post-treatment time points, coupled with progressive improvement over time, compared with vehicle control-treated eyes (Figure 5B and Figure S5B). Improvements could be observed both at dim flash intensities ≤0.1 cd·s/m^2^, representing rod-driven responses, and at brighter intensities >0.1 cd·s/m^2^, representing mixed rod–cone-driven responses [45]. Over time, there was a progressive amelioration in scotopic b-wave amplitudes in CompA-treated eyes, indicating functional regeneration (Figure 5C), which was not observed in vehicle control-treated eyes (Supplementary Figure S5C).

Under photopic conditions, brighter flashes of light elicit cone-driven electrical responses. As was the case under scotopic conditions, prior to MNU-induced retinal degeneration, both groups had similar b-wave amplitudes for all flash intensities recorded, whereas post-MNU, both groups had equally impaired b-wave amplitudes (Supplementary Figure S5D). Following four weekly IVT injections, CompA-treated eyes had significantly improved photopic b-wave amplitudes at all post-treatment time points compared with vehicle control-treated eyes (Figure 5D and Figure S5E). Over time, there was a progressive augmentation in photopic b-wave amplitudes for CompA-treated eyes but not vehicle control eyes (Supplementary Figure S5F,F′).

**Figure 6:**
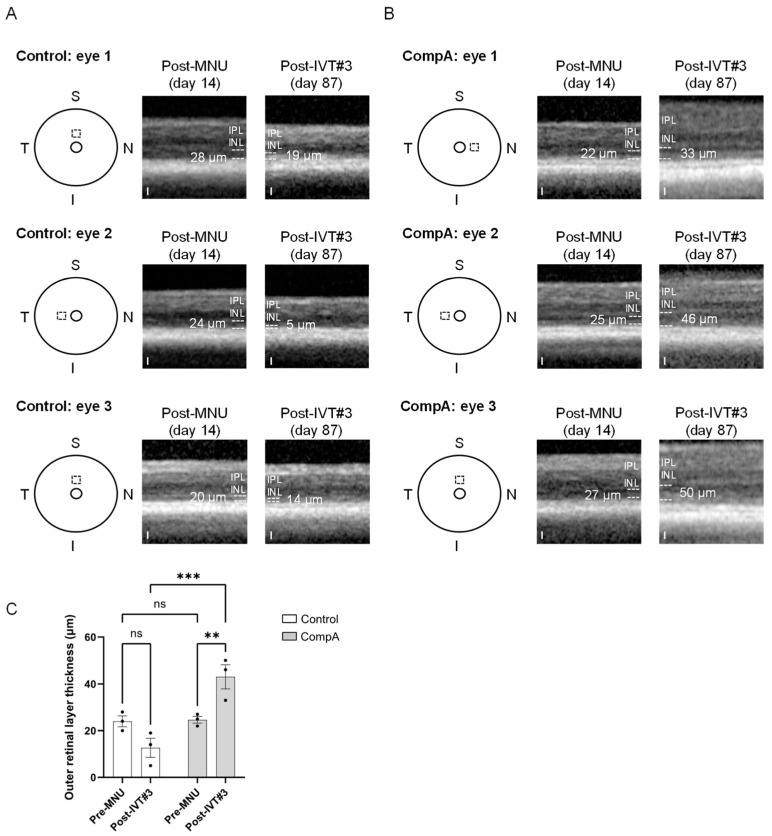
Adult C57BL/6J mice were given 35 mg/kg MNU IP to induce retinal degeneration. Two weeks after MNU, mice were given four weekly intravitreal (IVT) injections of CompA (0.14 µg per injection) or vehicle control. OCT imaging was performed 2 weeks following MNU administration and at 87 days following MNU (7.5 weeks post-treatment completion). (**A**) Representative OCT images were taken from three separate vehicle control group animals. Left: schematic representation indicating the location of the OCT scan beginning 400 µm from the optic nerve head (inner circle) in either the superior (S) or nasal (N) quadrant of the eye. The same location within individual eyes was imaged post-MNU (middle) and post-treatment (right). Dashed lines indicate the approximate borders of the outer retinal layers. White numbers indicate outer retinal layer (ORL; outer nuclear layer plus photoreceptor segments) thickness. IPL = inner plexiform layer, INL = inner nuclear layer. Note: the ORL along with other retinal layers could be difficult to distinguish in vehicle control-treated eyes at 87 days after MNU. No vehicle control-treated eyes (*n* = 3 eyes from 3 animals) had any restoration of the ORL post-treatment. Scale = 25 µm. (**B**) Representative OCT images from three separate CompA group animals. Left: schematic representation indicating the location of the OCT scan beginning 400 µm from the optic nerve head (inner circle) in either the superior (S), temporal (T), or nasal (N) quadrant of the eye (I = inferior). The same location within individual eyes was imaged post-MNU (middle) and post-treatment (right). Dashed lines indicate the approximate borders of the ORL. White numbers indicate ORL thickness. CompA-treated eyes with suitable OCT images (*n* = 3 eyes from 3 animals) show restoration of the ORL post-treatment. Scale = 25 µm. (**C**) Quantification of ORL thickness presented in (**A**,**B**). Only CompA-treated eyes had significantly improved ORL thickness compared to either vehicle controls or to the earlier post-MNU time point. ** *p* < 0.01, *** *p* < 0.001, *ns* = non-significant. Significance determined by two-way ANOVA with multiple comparisons. All results presented as mean ± SEM.

Under scotopic conditions, a-wave amplitudes become apparent in mice for flash intensities that elicit mixed rod–cone responses (see Figure 5A and Figure S4). Prior to MNU-induced retinal degeneration, both vehicle control and CompA groups had similar a-wave amplitudes for all flash intensities recorded under scotopic conditions, whereas post-MNU (and prior to IVT treatment), both groups had similarly impaired a-wave amplitudes due to MNU-induced retinal degeneration (Supplementary Figure S5G). Similar to scotopic and photopic b-waves, following four weekly IVT injections, CompA-treated eyes had significantly improved a-wave amplitudes at post-treatment time points compared with vehicle control-treated eyes (Figure 5E and Figure S5H). Over time, there was a progressive increase in scotopic a-wave amplitudes in CompA-treated eyes, whereas vehicle control a-wave amplitudes remained unchanged (Supplementary Figure S5I), suggesting that functional regeneration was driven by improvements in photoreceptor activity. CompA-treated eyes displayed increased thickness of outer retinal layers following treatment, as indicated by a comparison of OCT images taken prior to treatment initiation with images taken 7.5 weeks following treatment completion in individual eyes (Figure 6).

In brief, although both control and CompA group eyes had equally impaired vision prior to treatment, only CompA-treated eyes displayed functional regeneration over time. Based on ERG responses, the effect was driven by improvements in photoreceptor activity.

## 3. Discussion

One explanation for this regeneration of photoreceptor function is that CompA-mediated increases in RSPC proliferation were accompanied by improved differentiation and integration of functional photoreceptors. This notion is supported by (i) the increase in thickness of the ONL following treatment with CompA, (ii) the fact that treatment commenced following the completion of primary MNU-induced photoreceptor death, (iii) histological data suggestive of increased progenitor levels in the ONL following treatment, (iv) the presence of postnatal Msx1 lineage-derived cells in treated ONL, and (v) the magnitude and temporal profile of the sustained visual/retinal functional improvements. On the other hand, the continued existence of in vivo RSPCs in the adult mammalian eye remains controversial [8,9,10], and in our experiments, the numbers of detectable EdU and tdTomato-positive cells observed in the ONL are not sufficient to explain the observed functional regeneration, although this may correspond to our inability to properly detect all EdU (incomplete labeling, signal dilution, lack of EdU for the entire length of the experiment) and all tdTomato signals [11]. Therefore, another possibility is that functional regeneration may correspond to the recovery of pre-existing photoreceptors that did not die from the initial MNU insult. Finally, treatment may also result in a protective effect against any progressive photoreceptor death secondary to the initial MNU insult. However, regarding this last possibility, it is important to note that (i) treatment began following the primary wave of MNU-induced photoreceptor death, (ii) continued photoreceptor degeneration after two weeks had minimal impact on ONL thickness, (iii) OCT imaging indicates increased outer retinal layer thicknesses following treatment, and (iv) this alone cannot explain the magnitude of the functional improvements observed between the post-MNU and post-treatment time points. Given that photoreceptor regeneration, recovery, and survival may mutually reinforce one another, the restoration of visual functional observed here likely resulted from a combination of these possibilities.

It should be noted that the visual function experiments performed in this study utilized a chemically induced model, as opposed to a genetic model, of retinal degeneration. A single systemic dose of MNU leads to photoreceptors undergoing apoptosis which mechanistically resembles the cell death seen in inherited RP, coupled with ensuing electrophysiological and morphological changes also resembling RP [36]. The severity of degeneration varies depending on the dose of MNU administered, which can be exploited when designing experiments. On the other hand, MNU is a toxic DNA-alkylating agent that has the potential to produce non-specific or off-target effects. The MNU model also has the potential for variability in degenerative outcomes in cases where either the correct dose of MNU was not delivered or when animals have differential responsiveness to MNU. We offset this risk for variability by performing ONL quantifications one and two weeks following MNU, which demonstrates that both CompA and vehicle control groups had equivalent severity of degeneration prior to the initiation of treatment. Together, the present results suggest the potential for benefits in other inherited retinal degeneration models.

Elucidation of the cellular pathways responsible for mediating the beneficial effects of CompA remains ongoing. In vitro, we identified several kinases inhibited by CompA, which include p70S6K, ROCK1, and PKG. mTor signaling and ROCK signaling are both involved in regulating progenitor cell proliferation [26,46]. In addition, inhibition of these pathways, as well as inhibition of PKG signaling, have all been previously reported to lead to beneficial outcomes in models of retinal degeneration [25,47,48]. When using cell system-based biomarker profiling, we failed to identify any known compounds that reached the threshold for mechanistic similarity to CompA; however, the closest matches did include known ROCK and mTor inhibitors, which supports the idea that these pathways may be CompA’s cellular targets. In these cell systems, CompA was also shown to have tissue-remodeling activities, which included the modulation of TIMP levels. Within the degenerating retina, TIMP-1 modulation may influence photoreceptor tissue migration and organization and can exert beneficial effects in retinal degeneration models [49,50]. Future work will aim at confirming whether CompA modulates these pathways in vivo in the retina following intravitreal injection.

Regardless of the underlying cellular mechanisms, as proof of concept, we demonstrate how a single small molecule administered via intravitreal injection can promote the regeneration of visual function in an adult murine retinal degeneration model. The potential of an endogenous regeneration approach for retinal diseases may lead to a paradigm shift for patients who currently have no available treatment options.

## 4. Materials and Methods

In vitro kinase assay and kinome dendrogram. In vitro kinase inhibitory activities were measured using the Kinase-ProfilerTM (Eurofins Discovery Services; St. Charles, MO, USA) assay against 58 human kinases. The profiling of CompA was carried out using a fixed concentration of 5 μM. Visualization in the kinome tree was performed using the web-based tool KinMap [51]. Kinases inhibited by 50% or more are indicated in red with the circle size corresponding to the inhibition potency. The kinome dendrogram was adapted and is reproduced with courtesy of Cell Signaling Technology, Inc. (Danvers, MA USA; www.cellsignal.com; accessed on 1 December 2022).

Mouse lines. Young (8–10 week) or old (>36 week) adult male and female CD1 (Charles River, 022), C57BL6/J (Jackson Laboratories, 000664), or actinGFPrd1 mice (B5/EGFP; FVB.Cg-Tg(CAG-EGFP)B5Nagy/J; 003516, Jackson Laboratories) were used for sphere-forming assays. Given that the actinGFP mice were bred on an FVB background, all mice are homozygous were the Pde6brd1 mutation, which results in blindness by weaning age [52]. In rd1 mice, a spontaneous mutation in the rod β-subunit of phosphodiesterase (PDE) leads to rapid rod death (and secondary cone death) beginning by postnatal day 8. Msx1CreERT2;tdTomato mice were generated through the crossing of a previously generated C57BL/6J tamoxifen-inducible Msx1CreERT2 knock-in mouse line [53] with an Ai14 Rosa26-tdTomato Cre-reporter mouse line (007914, Jackson). In this line, tamoxifen is used to label (tdTomato) the subset of ciliary body cells that express Msx1 at the time of induction. All animal work was approved by the animal care committees at University of Toronto (AUP#20011120) and University Health Network in Toronto (AUP#6371), which operate in accordance with the Canadian Council on Animal Care. Animal husbandry was in accordance with the Association for Research in Vision and Ophthalmology (ARVO) Statement for the Use of Animals in Ophthalmic and Vision Research (room temperature: 18 °C to 23 °C; humidity: 40–60%; under 12/12 h light/dark cycle, except as required for ERG).

Compound preparation. In silico parameters were calculated using OpenMolecules software 5.5.0 (https://openmolecules.org/). For in vitro assays, stock solutions of Compound A (CompA, 10 mM) were diluted 1:1 in 100% DMSO to prepare 5 mM solutions. The compound was diluted 1:1000 in serum-free Neurocult Complete Mouse + Rat to a final concentration of 5 μM with 0.1% DMSO (*v*/*v*). For in vivo assays, CompA was prepared as a suspension (0.14 mg/mL) in Alcon’s balanced salt solution (BSS; 0065-0795-15). The vehicle control was Alcon’s BSS alone.

Mouse sphere-forming assay. In brief, this self-renewal assay involves dissociating the ciliary body, followed by plating the cells in culture for one week. The resulting structures are termed primary neurospheres. Subsequently, upon dissociating the primary neurospheres and continuing the cell culture for an additional week, the resulting structures are referred to as secondary neurospheres. This process is repeated, with the dissociation of secondary neurospheres and an additional week of cell culture, leading to the formation of tertiary neurospheres. Mouse eyes were collected immediately post-mortem and dissected as previously reported [54] to generate a single-cell suspension of ciliary epithelial cells. Cells were plated at clonal density (10 cells/μL) in serum-free Neurocult Complete Mouse + Rat Proliferation Media (StemCell Technologies, Vancouver, BC, Canada; 05702). After a week of growth, non-adherent primary neurosphere colonies were manually collected, dissociated in an enzyme mix containing Accutase (Sigma Aldrich, Oakville, ON, Canada; A6964), 0.7 mg/mL Hyaluronidase (Sigma, H2126), 0.5 mg/mL Collagenase 1 (Worthington, Lakewood, NJ, USA, LS004194), and 0.5 mg/mL Collagenase 2 (Worthington, LS004174), and plated as a single cell suspension (10 cells/μL) with CompA (5 μM) or 0.1% DMSO as the vehicle control for seven days in a 37 °C 5% CO_2_ incubator. Additionally, 10 cells/μL is sufficient dilution to ensure that clonally derived RSC colonies are generated [13]. Live cells were stained with 1 μM DRAQ5 (nuclear stain; Thermo Fisher, Mississauga, ON, Canada; 62251) and 0.1 μM CalceinAM (Sigma, 17783). Multi-well plates were imaged using an IN Cell Analyzer 6000 confocal microscope equipped with a Nikon 4X/NA 0.20 Plan Apo objective and a 2048 × 2048 sCMOS camera (GE Healthcare, Chicago, IL, USA). To count the number of objects, 3D images were acquired (10 z-sections, 150 nm spacing) for 12 fields per well. Z-stacks were collapsed using a maximum-intensity projection and analyzed using a custom routine for MATLAB2015b (MathWorks, Portola Valley, CA, USA). Object counts normalized to DMSO (normalized cell counts) were generated by dividing each value by the mean of the 0.1%DMSO condition for each experiment.

Human sphere-forming assay. The ciliary epithelium was dissected from a post-mortem human eye, and primary neurospheres were generated as previously described [13]. The neurospheres were then collected, resuspended in a 10% DMSO solution, and cryofrozen until use. Primary human neurospheres were thawed and allowed to recover in Neurocult Complete Human Proliferation Media (StemCell Technologies; 05751) for four days prior to dissociation in an enzyme mix containing 0.2 mg/mL kynurenic acid (FisherSci; H03031G), 1.33 mg/mL trypsin (Sigma; T1005-1G), 0.67 mg/mL hyaluronidase (Sigma; H2126), 0.5 mg/mL collagenase 1 (Worthington; LS004194), 0.5 mg/mL collagenase 2 (Worthington; LS004174), and 0.1 mg/mL elastase (Sigma; E1250). The resulting cell suspension was plated at clonal density (10 cells/μL) with CompA (5 μM) or 0.1% DMSO (*v*/*v*) as vehicle control. After one week of growth, cells were stained with DRAQ5 and imaged as described for mRSCs. Human eyes were obtained from the Eye Bank of Canada, and all experiments were approved by the University of Toronto research ethics board (REB#21094).

Differentiation and bulk RNA sequencing of cones from mRSC. A sphere-forming assay was performed as described above, with seven-day incubation in the presence of either CompA (5 μM) or 0.1% DMSO (*v*/*v*) as vehicle control. Subsequently, secondary neurospheres were plated on laminin-coated 24-well plates in differentiation medium containing 1% FBS and 50 ng/mL COCO (R&D Systems; 3047-CC) and incubated for 4–6 weeks. Differentiation medium was replaced twice weekly. Following differentiation, wells were either (i) fixed and used for immunocytochemistry or (ii) used for live-cell sorting.

For immunocytochemistry, one well from each biological replicate (*n* = 2) was fixed in 4% paraformaldehyde (AlfaAesar, Haverhill, MA, USA; J61899) for one hour at RT and then blocked in 10% FBS. Cells were incubated overnight at 4 °C with an anti-Cone Arrestin primary antibody (1:500; Sigma; AB15282) in 1% FBS, followed by washing in calcium- and magnesium-free (CMF)-DPBS. Cones were then incubated with Cy5-conjugated goat anti-rabbit (Invitrogen, Waltham, MA, USA; A10523) in 1% FBS for one hour at room temperature, followed by washing and counterstaining with 1 μg/mL Hoechst. To quantify cone differentiation, 12 fields per well were imaged using an IN Cell Analyzer 6000 confocal microscope equipped with a Nikon 4X/NA 0.20 Plan Apo objective and a 2048 × 2048 sCMOS camera (GE Healthcare) and analyzed using CellProfiler to identify nuclei and the number of cones per well.

For sorting, live cells were detached from the plate with 20 u/mL Papain (Worthington Chemicals; PAP), stained with DAPI (1 μg/mL) (Sigma; D9564), Alexa568-conjugated peanut agglutinin (10 μg/mL; Thermo Fisher; L32458), and sorted on either a BD Aria II or a BD Influx system. RNA extraction from at least 2500 cones/condition was performed using a kit (Qiagen, Singapore; 74106) as per the manufacturer protocol, and RIN (7.9-10.0) was checked to ensure RNA quality. A cDNA library was generated from total RNA using SMART-Seq V4 (Takara, Kusatsu, Japan). cDNA libraries were sequenced on an Illumina NovaSeq 6000 Sequencing System as 2 × 101-base-pair paired-end reads. Samples were mixed to obtain an average target depth of 70 million clusters that passed filtering. Reads shorter than 36 bp on either read1 or read2 were removed before mapping. Overall sequencing quality was examined using FastQC (v0.10.1). Alignment was performed with the STAR aligner (v2.6.0a) using mouse genome build mm10 and Gencode vM12 gene models. A previously generated cone-signature gene list of the top 40 differentially expressed (DE) genes between neurospheres and endogenous cones was used [30].

BioMap profiling. CompA was tested in the BioMAP Diversity PLUS panel at Eurofins (St. Charles, MO, USA), which consists of 12 primary human cell systems modeling vasculature, skin, lung, and inflammatory tissues (see Supplementary Table S1 for summary). Quantitative measurements of 148 biomarker activities were performed [55]. Biomarker activities were annotated when 2 or more consecutive concentrations changed in the same direction relative to vehicle controls, were outside of the significance envelope, and had at least one concentration with an effect size >20% (|log10 ratio| > 0.1). Antiproliferative effects were defined by an SRB or alamarBlue log10 ratio value <−0.1 from cells plated at a lower density.

For the unsupervised similarity analysis, CompA was compared to a reference database of >4500 BioMAP profiles to classify and identify the most similar profiles. This was performed using a combinatorial approach by filtering (Tanimoto metric) and ranking (BioMAP Z-Standard) the Pearson correlation coefficient between two profiles (BioMAP Z-Standard) [56]. Profiles are identified as having mechanistically relevant similarity if the Pearson correlation coefficient is ≥0.7.

Mechanism HeatMAP analysis provides a visualization of CompA at three concentrations together with 19 consensus mechanisms. The consensus profiles represent the average of multiple compounds from structurally distinct chemical classes. Profiles were calculated by averaging the values for each biomarker endpoint for all profiles selected to build the consensus mechanism profile [55]. Biomarker activities are colored red to represent increased protein expression and blue to represent decreased expression; white indicates levels that were unchanged. Darker shades of color represent greater change in biomarker activity relative to vehicle control.

Tamoxifen labelling of Msx1-positive cells. Tamoxifen (Sigma; T5648) was reconstituted in 90% sunflower oil (Sigma; S5007) and 10% ethanol and injected at 180 mg/kg intraperitoneally for four consecutive days.

Induction of retinal degeneration. To selectively degenerate retinal photoreceptors, we injected N-Nitroso-N-methylurea (MNU, Toronto Research Chemicals, Toronto, ON, Canada; M325815) intraperitoneally at either 45 mg/kg (Msx1CreERT2;tdTomato mice) or 35 mg/kg (C57BL6/J mice) in 8–11-week-old mice. In our hands, the severity and time course of MNU-induced degeneration was very consistent among different experiments, with 45 mg/kg leading to very severe ablation of the ONL in the lineage-tracing model and 35 mg/kg leading to mildly severe degeneration with slightly greater ONL preservation in C57BL6/J mice. While the 45 mg/kg was suitable for histological analysis, this dose was not used for functional testing because ONL ablation was too severe for the detection of residual function using the methods described below. MNU was reconstituted in 10% DMSO in CMF-DPBS.

Anesthesia and analgesia. For procedures requiring anesthesia and depending on the surgical procedure, mice were given either 5% isoflurane for induction (3% isoflurane for maintenance) via inhalation or intraperitoneal injection of 80 mg/kg ketamine and 10 mg/kg xylazine, with a maximum 50% top-up dose as required. Pupils were dilated with tropicamide (1% Mydriacyl^®^), and eyes were kept moist with either Systane^®^ gel or a 3% methylcellulose solution. To mitigate pain and distress as consequence of the procedures, mice received subcutaneous analgesia (Metacam, 1 mg/kg) prior to intravitreal injections.

**In vivo studies.** EdU (0.2 mg/mL) was provided ad libitum in 1% sucrose water for the first three weeks of the experiment to label proliferating cells. Daily water consumption was measured to ensure that all treatment groups consumed equivalent amounts of water. For intravitreal injections, mice were anesthetized as previously described; then, an intravitreal injection was performed. A bevelled 34G needle, attached to a 10 µL WPI Nanofil^®^ injector system (World Precision Instruments; IO-KIT), was inserted into the vitreous approximately 1 mm posterior of the limbus, taking care to avoid striking the lens or retina. Assuming that the volume of the mouse vitreous is 7 µL, 1 µL of CompA (400 µM, or 0.14 µg/injection) was injected to achieve a maximum vitreous concentration of 57 μM. Given the physical and chemical properties of CompA, together with its constitution as an ophthalmic suspension, we aimed for a dose approximately 100-fold greater than the in vitro half maximal effective concentration (EC50) and approximately 10-fold greater that the 5 uM concentration used in the majority of in vitro studies. CompA or vehicle control was injected at a rate of 3 µL/min, and the needle was maintained in the retrolental space for 30 s to permit pressure re-equilibration. A bilateral injection design was utilized for the following reasons: (i) it properly controls for the effect of 4 repeat IVT injections; (ii) it minimizes the number of animals required to obtain sufficient eyes suitable for functional analysis; and (iii) it helps control for any potential contralateral confounds.

Eye exclusions for functional analysis. Repeated intravitreal injections can lead to lens injury in mice, which can develop into cataracts over time, depending on the extent of the injury [57]. Other potential side effects of intravitreal injections, repeat anesthesia, or procedures involving repeat corneal contact can include blood in the vitreous, retinal detachment, and/or corneal opacity, which together may impair measures of visual function independently of photoreceptor health. All eyes were, therefore, assessed the day prior to each round of post-intravitreal (post-IVT) functional testing (post-IVT #1, post-IVT #2, post-IVT #3; see Figure 3). Each eye was scored on a 5-point scale by an experimenter blinded to the treatment condition. The scale consisted of: 1 = no detectable injection site or damage; 2 = visible injection site, minimal mark on lens, no lens discoloration; 3 = mild mark on lens localized near injection site or central lens without significant lens discoloration, mild localized cornea mark; 4 = visible lens trauma, focal lens discoloration at the injection site, displaced pupil, corneal opacity; 5 = obvious lens discoloration (cataract) covering a significant portion of the lens, significant amounts of blood in vitreous, significant corneal opacity (Supplementary Figure S6A). Presence of one (or more) of these characteristics resulted in that eye being assigned the highest associated score. Eyes scored 4 or above were excluded from subsequent functional analysis. In the initial assessment prior to the post-IVT #1 time point, 14 vehicle control-treated eyes and 14 CompA-treated eyes scored below 4 and were deemed suitable for visual function testing. The average score for all CompA eyes was 3.458, and the average score for all vehicle control eyes was also 3.458, indicating that the two groups were equally affected by IVT (Supplementary Figure S6B).

Optical coherence tomography (OCT). Mice were anesthetized, and pupils dilated, prior to image acquisition with a Heidelberg Spectralis OCT + HRA coupled with a mouse adaptor lens. Vertical (superior–inferior axis) and horizontal (nasal–temporal axis) line scans were performed near the optic nerve head. Two weeks following MNU-induced retinal degeneration, OCT imaging was performed, and ONL thickness measurements were made at 400 µm from the optic nerve head in each quadrant of the retina and then averaged. All quantification was performed by an experimenter blinded to the treatment condition. Quantifiable OCT images could not be reliably obtained following treatment completion due to technical challenges in obtaining adequate OCT scans; however, a small subset of eyes had OCT images taken 87 days following MNU (7.5 weeks following treatment condition).

Visual acuity and contrast sensitivity. The innate reflexive optomotor response occurs when a mouse’s visual field is rotated and can be used to determine visual thresholds using the OptoMotry device (CerebralMechanics; OptoMotry VR 1.7.7) as described previously [44,58]. Only temporal-to-nasal-directed motion drives the response, which allows each eye to be measured independently by changing the direction of motion [58]. Briefly, an operator blinded to the treatment condition placed a mouse on a raised platform within a virtual cylinder which was rotated in either the clockwise or counterclockwise direction at 12 degrees/second. For visual acuity, a randomized protocol design with a simple staircase psychophysical method was used at 100% contrast. Visual acuity is reported as the maximum spatial frequency (cycles/degree) at which head movements can still reliably be elicited. For visual stimuli, a contrast of 100% means black/white patterns. The OptoMotry device can also be used to determine the minimum contrast threshold (%) able to reliably drive head movements. Contrast sensitivity is the reciprocal of the contrast threshold. Contrast thresholds were determined at two user-defined spatial frequencies (0.189 and 0.289 cycles/degree). Contrast sensitivity was not measured pre-MNU given that the two user-defined spatial frequencies (0.189 and 0.289 cycles/degree) had not yet been established. The minimum contrast threshold (%) able to reliably drive head movements was determined. Contrast sensitivity is the reciprocal of the contrast threshold. Normal adult mouse contrast sensitivity values for these spatial frequencies should approximate 20–25 [58].

Electroretinogram. ERG recordings were performed by an experimenter blinded to the treatment conditions. ERG light stimulation and recordings were performed using a Diagnosys Espion E3 rodent ERG device with a ColorDome Ganzfield stimulator (Diagnosys) and Espion V6 software. All recordings were performed in the dark with red light for illumination. Prior to recordings, mice were dark-adapted overnight. Throughout the procedure, the body temperature was maintained at 38 °C with a heated platform (Diagnosys). The corneas were kept moist with a thin layer of Systane^®^ gel. Simultaneous bilateral recordings were performed using gold loop electrodes placed on the cornea of each eye. A subdermal platinum reference electrode was placed between the eyes, and a ground platinum electrode was placed at the base of the tail. Testing was performed by a blinded operator using a modified version of a previously published protocol [44]. Briefly, light stimulation consisted of brief pulses of white light. Scotopic recordings were first performed in mice that had been dark-adapted overnight. A total of 5–10 recordings per stimulus intensity were averaged, and the a- and b-wave amplitudes were determined by the software used. Interstimulus intervals were 10–30 s, with increased values at brighter flash intensities. The following stimulus conditions (flash intensities) were used: 0.00025 cd·s/m^2^, 0.0025 cd·s/m^2^, 0.025 cd·s/m^2^, 0.25 cd·s/m^2^, 2.5 cd·s/m^2^, 5 cd·s/m^2^, and 10 cd·s/m^2^. Mice were then light-adapted with 30 cd·s/m^2^ background illumination for 600 s. Photopic tests were performed in the presence of 30 cd·s/m^2^ background illumination. Up to 30 recordings per flash intensity were averaged, and the amplitude of the a- and b-waves were determined by the software used. The following stimulus intensities were used: 0.15 cd·s/m^2^, 1.25 cd·s/m^2^, 5 cd·s/m^2^, 10 cd·s/m^2^, and 25 cd·s/m^2^.

Immunohistochemistry. Eyes were fixed in 4% paraformaldehyde (AlfaAesar; J61899) for four hours and cryoprotected overnight at 4 °C in a 30% sucrose solution before embedding in Neg-50™ Frozen Section Medium (Thermo Fisher, Mississauga, Canada; 6502). Sections of 10 µm were generated using a Leica CM2050S cryostat. Sections were stained for EdU using the Click-IT™ EdU Proliferation Kit (Thermo Fisher; C10337) as per the manufacturer’s protocol. For antibody labelling, sections were permeabilized in either 0.3% TritonX-100 or 0.1% Tween 20 for 20 min, blocked in 10% serum for one hour, and incubated overnight with primary antibodies at 4 °C diluted in 1% serum. For Rax staining, heat-induced antigen retrieval was performed in 10 mM citric acid (pH 6) prior to blocking. Following primary incubation, the sections were incubated for one hour with secondary antibodies diluted in 1% serum for one hour at room temperature and counterstained with either 1 μg/mL Hoechst or 1 μM DRAQ5 for 10 min. Sections were mounted with Prolong^®^ Glass anti-fade mounting medium (Invitrogen; P36984). Note that the serum used for each staining was procured from the same species as the secondary antibodies were raised in.

The primary antibodies used were anti-Otx2, 8 µg/mL (R&D Systems; BAF1979); anti-Cone Arrestin, 1 μg/mL (Sigma; AB15282); anti-Rax, 4 µg/mL (Santa-Cruz; sc-271889); anti-Rhodopsin, 0.4 μg/mL (Sigma; MAB5316); anti-PKCα (R&D Biosystems; AF5340); and anti-tdTomato, 2 µg/mL (Rockland; 600-401-379). The secondary antibodies used were Cy5-goat anti-rabbit (Invitrogen; A10523), Alexa488-goat anti-mouse (Invitrogen; A32723TR), and Alexa647-donkey anti-goat (Invitrogen; A21447), all diluted 1:500 in 1% serum. Slides were imaged using a Nikon A1 confocal microscope or with a Zeiss Z.1 Axioscan slide scanner.

Immunohistochemistry image analysis. To determine the proportion of Msx1+ cells that were proliferating within the ciliary body (Supplementary Figure S6C), the number of Msx1+, EdU+, and Msx1/EdU double-positive cells in each ciliary body were counted in a minimum of three sections (10 µm thick) per eye and averaged. To ensure that the animal had received an adequate supply of EdU to label proliferating cells within the eye, lens capsule cells, a known source of proliferating cells within the eye, were inspected to confirm positive staining. The number of Msx1/EdU double-positive cells was normalized to the number of Msx1+ cells.

Nuclear Otx2 expression is a marker of mature bipolar and early photoreceptor-committed progenitors [37,38,39]. Otx2 expression dissipates to a perinuclear staining pattern as photoreceptors mature [40]. Otx2 progenitors in the ONL were scored by counting the number of nuclear-stained Otx2+ cells within 200 µm of the neural tip of the retina. A minimum of two sections per eye were analyzed by an experimenter blinded to the treatment condition and averaged.

To determine ONL thickness, five equidistant points across the retinal section were measured using the line tool in Zeiss Blue software and then averaged (Supplementary Figure S6D). All quantifications and selection of regions were performed by experimenters blinded to the treatment condition. Sections from the approximate central retina (i.e., sections either near or containing the optic nerve) were used for quantification. To determine the location of the measurements, for each section, using the nuclear channel and beginning near the periphery of the ONL, the length of the ONL was traced and subdivided into 4 equidistant quadrants. A measurement was made at the edges of each quadrant, leading to 5 equidistant points. A minimum of three sections (10 µm thick) were scored per eye and averaged for ONL thickness quantification. To determine the number of cones per 200 µm length of ONL, the number of cone arrestin+ cells were counted along 10 µm thick cryosections. Two sections per eye were scored and averaged for cone quantification.

To aid in visualization and verify the specificity of the tdTomato signal within the retina, some sections were stained with an anti-tdTomato antibody. A minimum of two sections per eye were manually inspected to identify tdTomato+ cells within the retina.

## Data Availability

The data that support the findings of this study are available from the corresponding author upon reasonable request.

## Supplementary Materials

The following supporting information can be downloaded at: https://www.mdpi.com/article/10.3390/ijms25031521/s1.
