# Supplementary material for: Small-Molecule-Directed Endogenous Regeneration of Visual Function in a Mammalian Retinal Degeneration Model
Source: Int J Mol Sci. 2024 Jan 26;25(3):1521. doi: 10.3390/ijms25031521 (PMC10855388; doi:10.3390/ijms25031521)
Supplement: Supplementary file 1 [file ijms-25-01521-s001.zip › ijms-2697662-supplementary.pdf]

Supplementary figures

Supplementary Figure S1

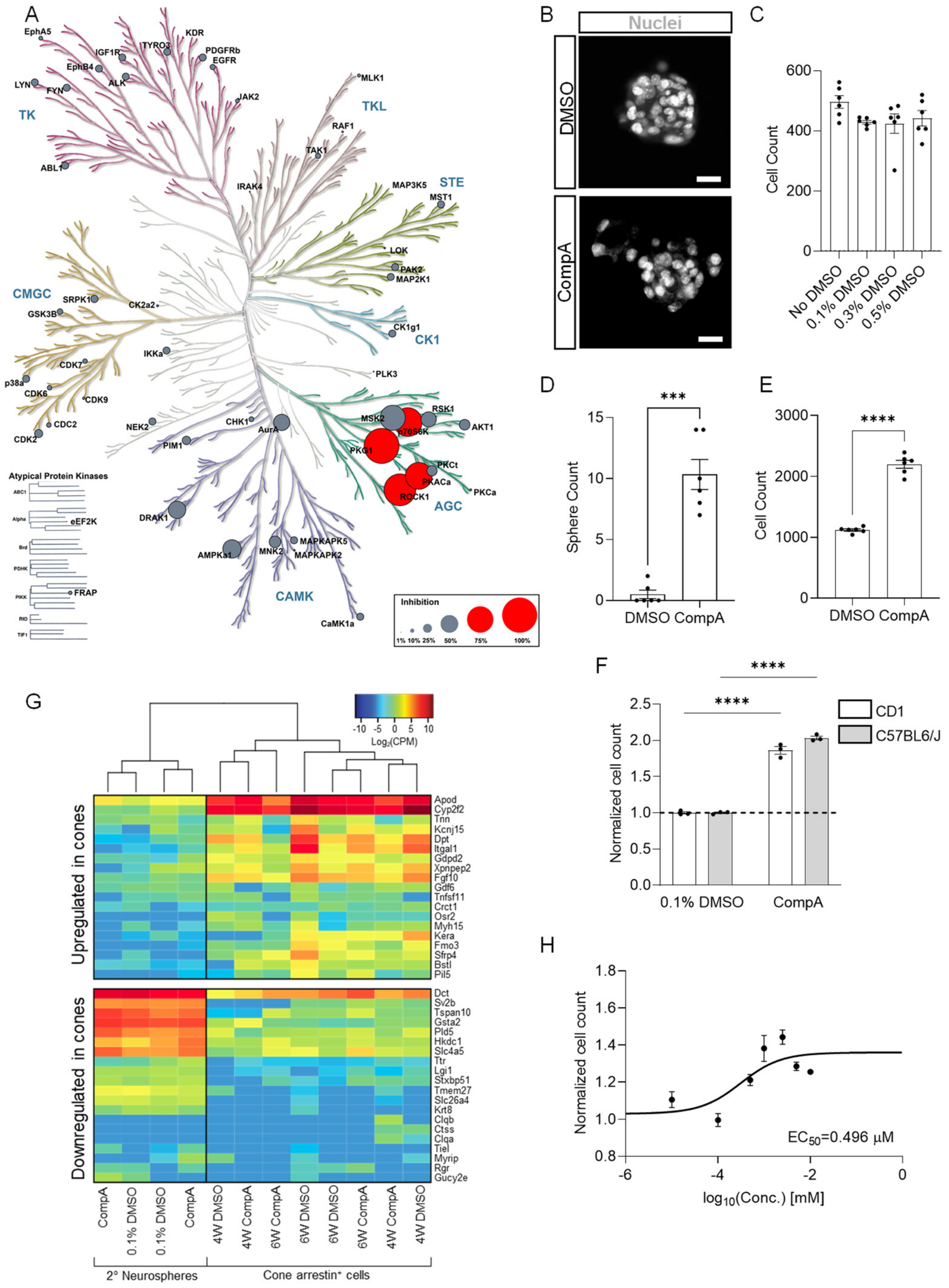

Supplementary Figure S1. A, *Map of CompA (5 µM) inhibitory activities tested on a panel of 58 kinases and visualized on a Kinase dendrogram. CompA inhibition potency (%) correspond to the size of circle for each kinase annotated in the dendrogram. Inhibitory potency of 74% or less appears in grey, inhibitory potency of 75% or more appear in red. The kinome dendrogram was adapted and is reproduced courtesy of Cell Signaling Technology, Inc.* ***B***, *In the mouse RPSC phenotypic assay, the size of nuclei (grey) within secondary spheres is the same between treatment conditions.* ***C***, *Primary ciliary epithelium were dissected and primary neurospheres were generated. After dissociation, a single cell suspension was plated with various concentrations of DMSO. There was no significant effect of DMSO on the proliferation of RSPC in vitro in concentrations up to 0.5%,*

five times the concentration used in the phenotypic assays. ***D-E****, Tertiary spheres were generated from (5 µM) CompA- or (0.1%) DMSO-treated secondary spheres. CompA or DMSO treatment was continued during the generation of tertiary spheres.* ***D****, Continuous treatment with CompA (5 µM) increases the formation of tertiary spheres over controls.* ***E****, Cell counts within (5 µM) CompA-treated tertiary spheres were increased over (0.1%) DMSO-treated tertiary spheres.* ***F****, Proliferation can also be increased in mouse RSPC isolated from aged mice (>36 weeks-old). n = 3 for each; *** p < 0.001, ****p < 0.0001; two-way ANOVA with corrections for multiple comparisons.* ***G****, Heatmap of differential expression of cone signature genes in CompA (5 µM) or (0.1%) DMSO treated secondary neurospheres (RSPC) and CompA- or DMSO-derived cones. The transcriptomes of DMSO- and CompA-derived cones cluster together and resemble endogenous cones.* ***H****, rd1 derived spheres were exposed to seven different concentrations of CompA for one week. Secondary neurosphere cell counts were normalized to controls, and the EC50 was calculated using a non-linear regression model (four parameters). The EC50 of CompA is 0.496 µM. Scale bars= 20 µm. All results presented as mean ± SEM.*

Supplementary Figure S2

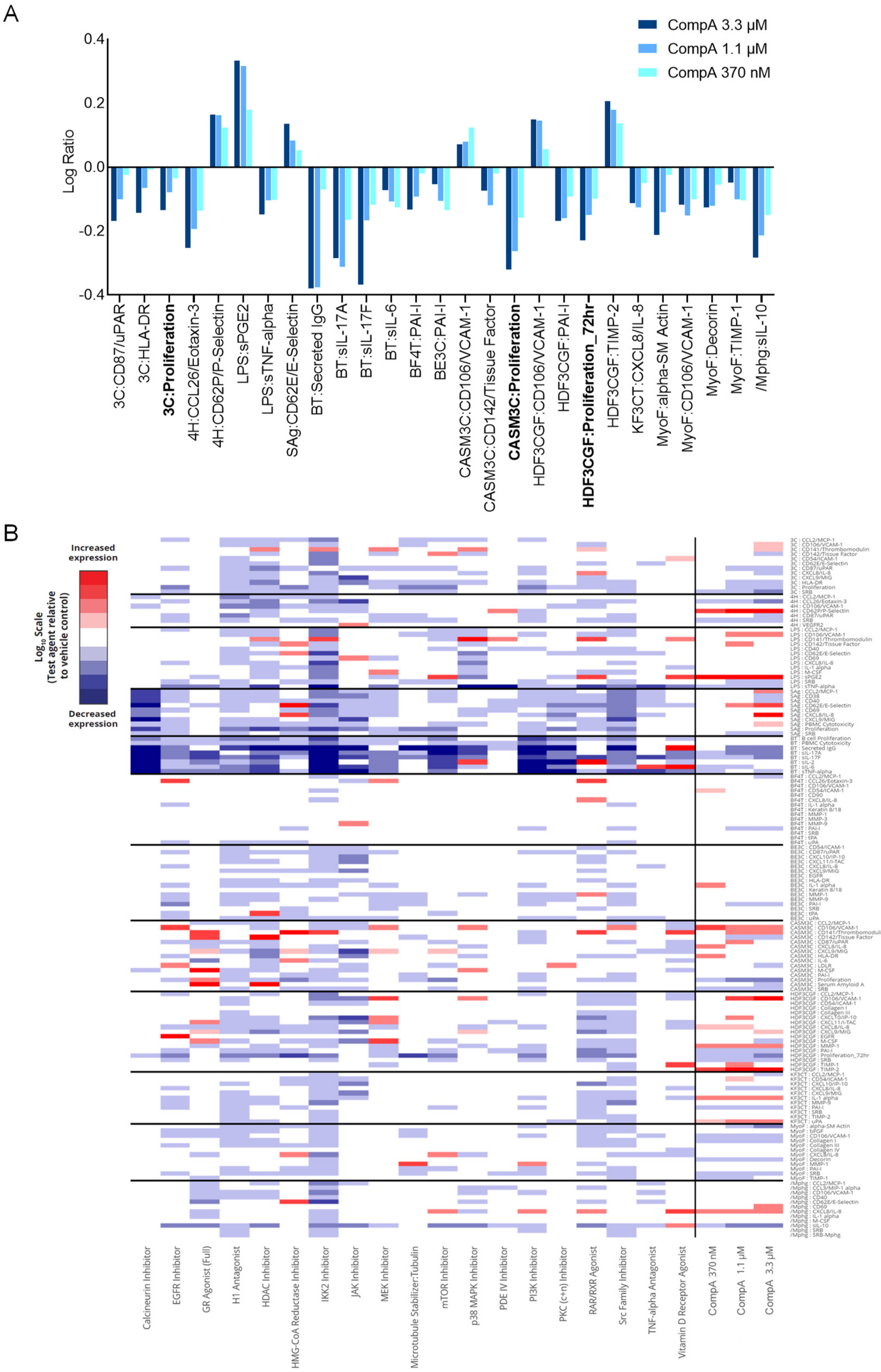

***Supplementary Figure S2. A**, Effects of three concentrations of CompA in a 12-system biomarker-based profiling panel (BioMAP® Diversity PLUS® - Eurofins Discovery Services). The Y-axis represents a log-transformed ratio of the biomarker readouts for the CompA-treated sample (n = 1) over vehicle controls (average of ≥ 6 vehicle controls from the same plate). The X-axis consists of annotated biomarkers (system name followed by biomarker name; see Appendix A - Supplementary*

Table 1 for description). Proliferation readouts are indicated in bold. ***B****, Mechanism heatmap analysis consisting of 148 biomarker readouts (rows) of 19 consensus mechanism class profiles (columns) together with the CompA profile at three concentrations. Horizontal grey lines separate the 12 systems of the panel (Supplementary Table S1), while the vertical grey line separates CompA from the 19 consensus mechanism profiles. Biomarker activities outside of the significance envelope are red if protein levels are increased, blue if protein levels are decreased and white if levels are not significantly changed. Darker shades of color represent greater change in biomarker activity relative to vehicle control.*

Supplementary Figure S3

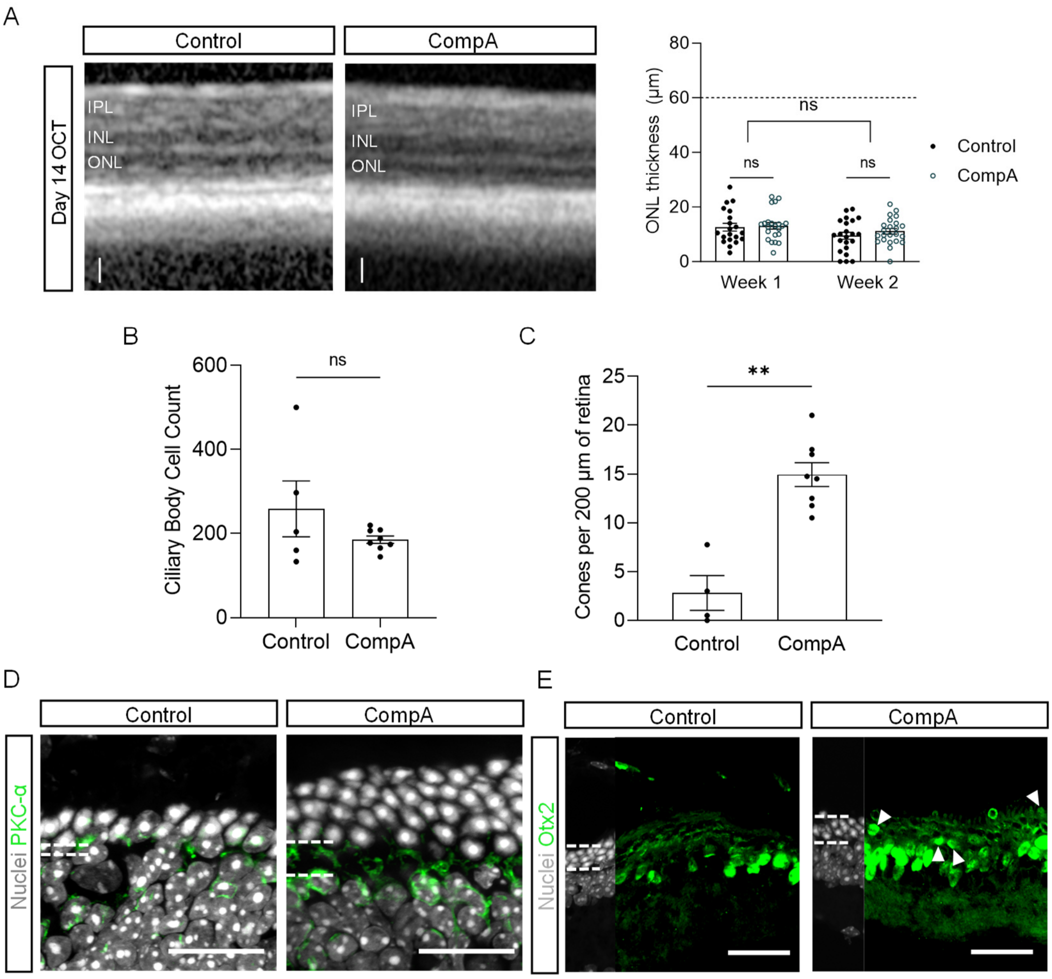

Supplementary Figure S3. A*, representative OCT images of a control group (top) and an CompA group (bottom) retina. White arrows indicate the ONL layer. Right: quantification of the outer nuclear layer (ONL) thickness in the central retinal comparing control group to CompA group (n = 22 for control and n = 23 for CompA; unpaired t-test; ns = not significant). OCT images were taken one week and two weeks after induction of retinal degeneration by MNU (35 mg/kg). There was no significant difference (ns) in ONL thickness between treatment groups prior to intervention. Dotted line represents approximate size of a normal ONL.* ***B****, There was no significant difference (ns) between treatment groups in the number of nuclei within each ciliary body. Ciliary bodies that displayed gross staining or anatomical abnormalities were excluded from analysis* ***C****, Quantification of the number of cones per 200 µm length of ONL in 10 µm thick cryosections. **p < 0.01; Student's t-tests with Welch's correction. All data are represented as mean ± SEM.* ***D****, Representative images of PKC-α (green) staining of rod ON bipolar cells together with a nuclei stain (Hoechst; grey). CompA-treated retinas have interneuron processes that extend into the outer plexiform layer (OPL), while control-treated retina have fewer bipolar processes displaying*

synapse-like morphology in the OPL. Dashed lines indicate border of the OPL. ***E****, Treatment with CompA increases the number of cells within the outer nuclear layer (ONL) of the neural tip that are positive for retinal progenitor markers. White dashed lines indicate approximate border of the ONL. White arrows indicate Otx2 (green) localized in the nucleus. Scale bars= 50 µm. All data are represented as mean ± SEM.*

Supplementary Figure S4

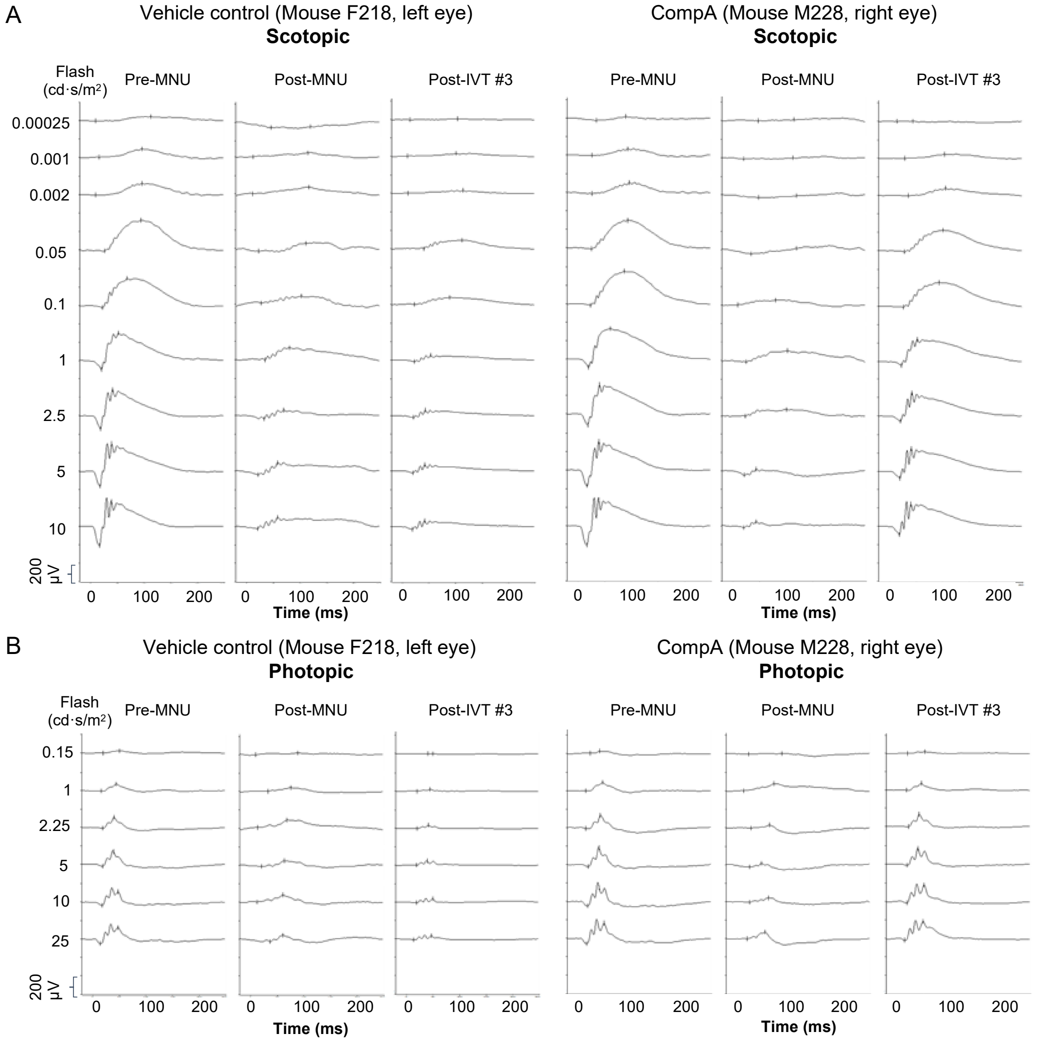

Supplementary Figure S4. A,B*. C57BL6/J mice were given 35 mg/kg MNU via IP to induce retinal degeneration. Two weeks after MNU, mice were given four weekly IVT injections of CompA or vehicle control. ERG recordings were performed prior to MNU administration (Pre-MNU), following MNU but prior to treatment (Post-MNU), and at various time points following treatment (Post-IVT #1 - #3).* ***A****. Left: representative ERG traces of a vehicle control-treated eye (F218, left eye) taken under scotopic conditions. Right: Representative ERG traces of an CompA-treated eye (M228, right eye) taken under scotopic conditions.* ***B****. After 10 min of light adaptation, photopic ERGs were performed. Left: representative ERG traces of a vehicle control-treated eye (F218, left eye) taken under photopic conditions. Right: representative ERG traces of a CompA-treated eye (M228, right eye) taken under photopic conditions.*

Supplementary Figure S5

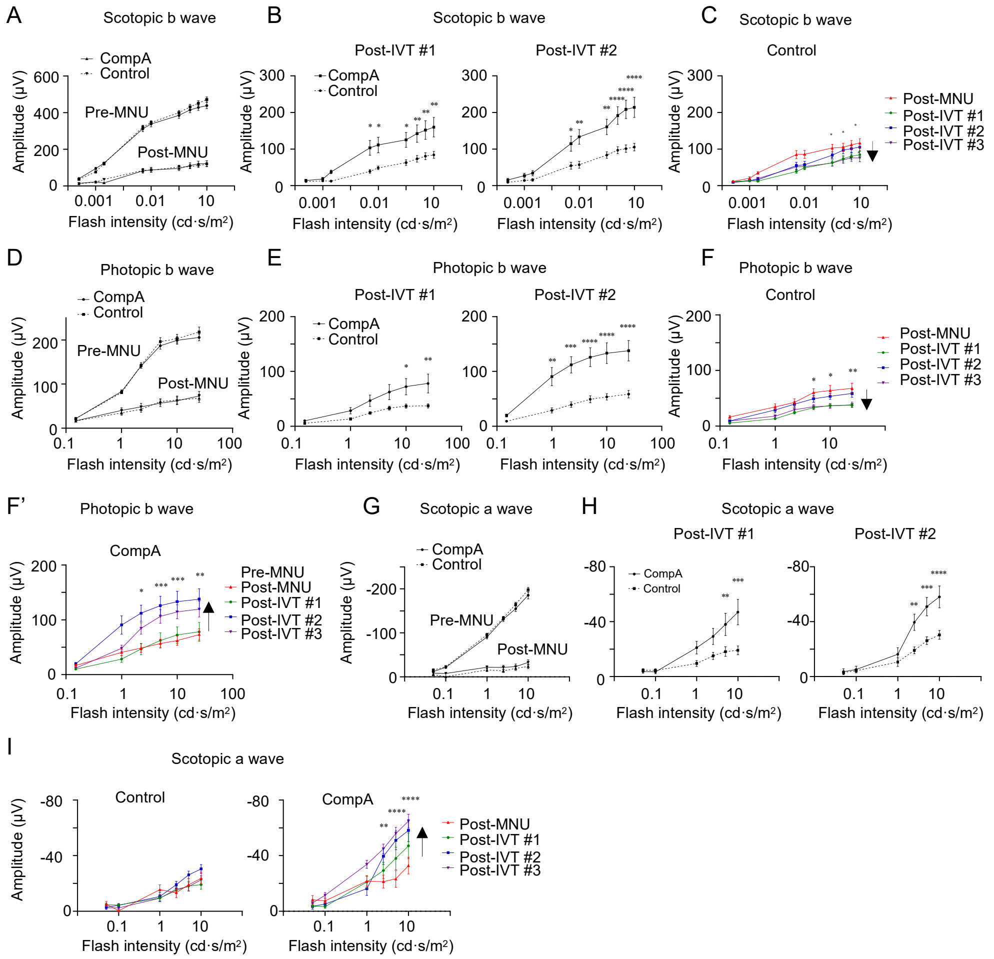

***Supplementary Figure S5. A**, There were no significant differences in scotopic b-wave amplitudes between control and CompA eyes at Pre-MNU (left; n = 18 eyes from 9 mice for control and n = 24 eyes from 12 mice for CompA) or Post-MNU (right; n = 22 eyes from 11 mice for both). **B**, CompA-treated retina had significantly improved scotopic b wave amplitudes compared to controls at Post-IVT #1 (left; n = 14 eyes for both; 10 animals for control and 9 animals for CompA) and Post-IVT #2 (right; n = 14 eyes for both; 10 animals for control and 9 animals for CompA) time points. **C**, Over time there was a decline in scotopic b wave amplitudes in control treated eyes between Post-MNU and Post-IVT #3 (n = 22 and 13 eyes respectively; * = Post-MNU vs Post-IVT #3). **D**, There were no significant differences in photopic b-wave amplitudes between control and CompA eyes at Pre-MNU (left; n = 18 eyes for control and n = 24 for CompA) or Post-MNU (right; n = 22 for both). **E**, CompA-treated retina had significantly improved photopic b wave amplitudes compared to controls at Post-IVT #1 (left; n = 14 for both) and Post-IVT #2 (right; n = 14 for both). **F-F'**, Over time photopic b wave amplitudes declined in control treated eyes (left) but increased in CompA-treated eyes between Post-MNU and Post-IVT #3 (Post-MNU n = 22 for both control and CompA; Post-IVT #3 n=13 eyes from 9 animals for controls and n=12 eyes from 8 animals for CompA; * = Post-MNU vs Post-IVT #3). **G**, There were no significant differences in a- wave amplitudes between control and CompA eyes Pre-MNU or Post-MNU. **H**, CompA-treated retina had significantly improved a wave amplitudes compared to controls at Post-IVT #1 (left; n = 14 for both) and Post-IVT #2 (right; n = 14 for both). **I**, Over time, a wave amplitudes in controls (left) remained unchanged, whereas a wave amplitudes significantly improved in CompA-treated eyes (right) between Post-MNU and Post-IVT #3 (Post-MNU n = 22 for controls and for CompA; Post-IVT #3 n = 13 for controls and n = 12 for CompA; * = Post-MNU vs Post-IVT). For all: *p < 0.05, ** p < 0.01, *** p < 0.001, **** p < 0.0001; two-way ANOVA followed by Sidak's multiple comparison test, data presented as mean ± SEM. Arrow indicates direction of change.*

Supplementary Figure S6

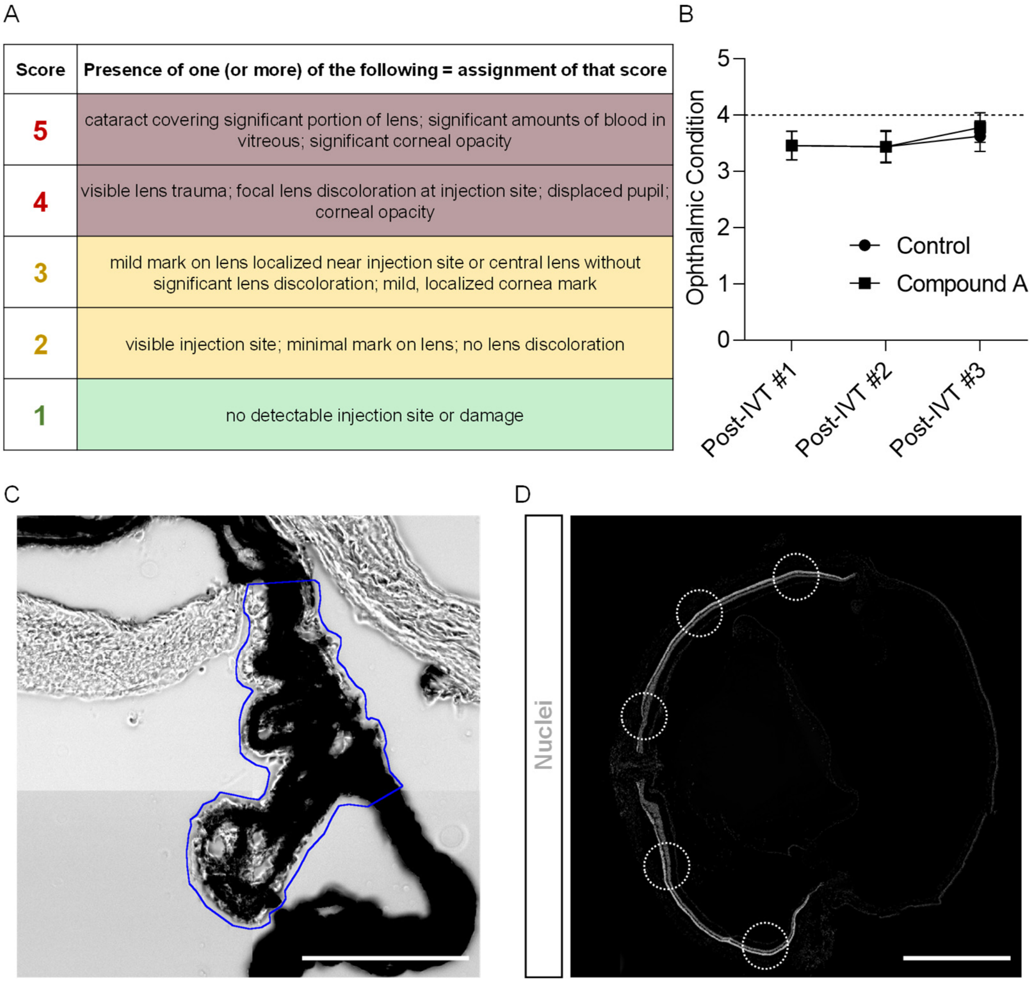

Supplementary Figure S6. A-B*, The condition of each eye was scored prior to the initiation of functional tests on a five-point system. Eyes that scored above four were excluded from subsequent functional testing. Eye condition remained stable after the interventional phase of the experiment. There was no difference in eye health between treatment conditions at any time point.* ***C****, The ciliary body is defined morphologically (blue line) as the region posterior to the iris, anterior to the iris, and between the vitreous and sclera. Analyses to determine RSPC proliferation were performed in this region.* ***D****, To determine ONL thickness, five points equidistant across the retina were measured using the line tool in Zen Blue (Zeiss). Scale bars= (C) 50 µm; (D) 1mm.*

Supplementary Table S1

Supplementary Table S1*: The 12 systems comprising the BioMAP® Diversity PLUS® phenotypic profiling panel (Eurofins Discovery Services). Listed are the names of the 12 systems, the primary human cell types used, the disease/tissue relevance, a description of the system and the biomarkers measured in each system. PBMC = peripheral blood mononuclear cells; COPD = chronic obstructive pulmonary disease.*

Available Systems & Translational Biomarker Readouts				
**System**	**Human Primary Cell Types**	**Diseases/Tissues Modeled**	**Description**	**Protein Biomarker Readouts**
**3C**<br>Th1 Vasculature	Venular endothelial cells	Cardiovascular Disease, Chronic Inflammation	The Th1 Vasculature (3C) system models vascular inflammation of the Th1 type, an environment that promotes monocyte and T cell adhesion and recruitment and is anti-angiogenic. This system is relevant for chronic inflammatory diseases, vascular inflammation and restenosis.	MCP-1, VCAM-1, TM, TF, ICAM-1, E-selectin, uPAR, IL-8, MIG, HLADR, Proliferation, SRB
**4H**<br>Th2 Vasculature	Venular endothelial cells	Asthma, Allergy, Autoimmune Disease, Atopic Disease	The Th2 Vasculature (4H) system models vascular inflammation of the Th2 type, an environment that promotes mast cell, basophil, eosinophil, T and B cell recruitment and is proangiogenic. This system is relevant for diseases where Th2-type inflammatory conditions play a role such as allergy, asthma, and ulcerative colitis.	MCP-1, Eotaxin-3, VCAM-1, P-selectin, uPAR, SRB, VEGFR2
**LPS**<br>Monocyte Activation	PBMC/Venular endothelial cells	Cardiovascular Disease, Chronic Inflammation	The Monocyte Activation (LPS) system models chronic monocyte activation and vascular inflammation. This system is relevant to inflammatory conditions where monocytes play a key role including atherosclerosis, restenosis, rheumatoid arthritis, other chronic inflammatory conditions and metabolic diseases.	MCP-1, VCAM-1, TM, TF, CD40, E-selectin, CD69, IL-8, IL-1α, M-CSF, sPGE2, SRB, sTNFα
**SAg**<br>T Cell Activation	PBMC/Venular endothelial cells	Autoimmune Disease, Chronic Inflammation	The T Cell Activation (SAg) system models vascular inflammation and T cell activation. This system is relevant to inflammatory conditions where T cells play a key role including organ transplantation, rheumatoid arthritis, psoriasis, Crohn's disease and multiple sclerosis.	MCP-1, CD38, CD40, E-selectin, CD69, IL-8, MIG, PBMC Cytotoxicity, Proliferation, SRB
**BT**<br>B and T Cell Autoimmunity	B cells/PBMC	Autoimmune Disease, Inflammation	The B and T Cell Autoimmunity (BT) system models T cell dependent B cell activation and class switching as would occur in a germinal center. This system is relevant for diseases and conditions where B cell activation and antibody production are relevant. These include autoimmune disease, oncology, asthma and allergy.	B cell Proliferation, PBMC Cytotoxicity, Secreted IgG, sIL-17A, sIL-17F, sIL-2, sIL-6, sTNFα
**BF4T**<br>Lung Disease	Bronchial epithelial cells/Dermal fibroblasts	Asthma, Allergy, Lung Inflammation, Atopic Disease	The Lung Disease (BF4T) system models lung inflammation of the Th2 type, an environment that promotes the recruitment of eosinophils, mast cells and basophils as well as effector memory T cells. This system is relevant for allergy and asthma, pulmonary fibrosis, as well as COPD exacerbations.	MCP-1, Eotaxin-3, VCAM-1, ICAM-1, CD90, IL-8, IL-1α, Keratin 8/18, MMP-1, MMP-3, MMP-9, PAI-1, SRB, tPA, uPA
**BE3C**<br>Lung Inflammation	Bronchial epithelial cells	Lung Inflammation, COPD	The Lung Inflammation (BE3C) system models lung inflammation of the Th1 type, an environment that promotes monocyte and T cell adhesion and recruitment. This system is relevant for sarcoidosis and pulmonary responses to respiratory infections.	ICAM-1, uPAR, IP-10, I-TAC, IL-8, MIG, EGFR, HLA-DR, IL-1α, Keratin 8/18, MMP-1, MMP-9, PAI-1, SRB, tPA, uPA
**CASM3C**<br>Cardiovascular Disease	Coronary artery smooth muscle cells	Cardiovascular Inflammation, Restenosis	The Cardiovascular Disease (CASM3C) system models vascular inflammation of the Th1 type, an environment that promotes monocyte and T cell recruitment. This system is relevant for chronic inflammatory diseases, vascular inflammation and restenosis.	MCP-1, VCAM-1, TM, TF, uPAR, IL-8, MIG, HLA-DR, IL-6, LDLR, M-CSF, PAI-1, Proliferation, SAA, SRB
**HDF3CGF**<br>Wound Healing and Inflammation	Dermal fibroblasts	Fibrosis, Chronic Inflammation, Wound Healing, Tissue Remodeling, Matrix Modulation	The Wound Healing, Fibrosis and Inflammation (HDF3CGF) system models wound healing and matrix/tissue remodeling in the context of Th1-type inflammation. This system is relevant for various diseases including fibrosis, rheumatoid arthritis, psoriasis, as well as stromal biology in tumors.	MCP-1, VCAM-1, ICAM-1, Collagen I, Collagen III, IP-10, I-TAC, IL-8, MIG, EGFR, M-CSF, MMP-1, PAI-1, Proliferation, SRB, TIMP-1, TIMP-2
**KF3CT**<br>Psoriasis and Dermatitis	Keratinocytes/ Dermal fibroblasts	Psoriasis, Dermatitis, Skin Biology	The Psoriasis and Dermatitis (KF3CT) system models cutaneous inflammation of the Th1 type, an environment that promotes monocyte and T cell adhesion and recruitment. This system is relevant for cutaneous responses to tissue damage caused by mechanical, chemical, or infectious agents, as well as certain states of psoriasis and dermatitis.	MCP-1, ICAM-1, IP-10, IL-8, MIG, IL-1α, MMP-9, PAI-1, SRB, TIMP-2, uPA
**MyoF**<br>Fibrosis	Lung fibroblasts	Fibrosis, Chronic Inflammation, Matrix Remodeling	The Fibrosis (MyoF) system models the development of pulmonary myofibroblasts and is relevant to respiratory disease settings as well as other chronic inflammatory settings where fibrosis occurs, such as rheumatoid arthritis.	a-SM Actin, bFGF, VCAM-1, Collagen I, Collagen III, Collagen IV, IL-8, Decorin, MMP-1, PAI-1, TIMP-1, SRB
**lMphg**<br>Macrophage Activation	Venular endothelial cells/ Macrophages	Cardiovascular Inflammation, Restenosis, Chronic Inflammation	The Macrophage Activation (/Mphg) system models chronic inflammation of the Th1 type and macrophage activation responses. This system is relevant to inflammatory conditions where monocytes play a key role including atherosclerosis, restenosis, rheumatoid arthritis, and other chronic inflammatory conditions.	MCP-1, MIP-1α, VCAM-1, CD40, E-selectin, CD69, IL-8, IL-1α, M-CSF, sIL-10, SRB, SRB-Mphg

Supplementary Table S2

Supplementary Table S2: *Top BioMAP Reference Database Matches for each concentration of CompA. A table of the top three similarity matches from an unsupervised search of the BioMAP Reference Database of > 4,500 agents. The similarity between agents is determined using a combinatorial approach that accounts for the characteristics of BioMAP profiles by filtering (Tanimoto metric) and ranking (BioMAP Z-Standard) the Pearson's correlation coefficient between two profiles. Profiles are identified as having mechanistically relevant similarity if the Pearson's correlation coefficient is ≥ 0.7.*

CompA	Database Match	BioMAP Z-Standard	Pearson's Score	# of Common Readouts	Mechanism Class
370 nM	Torin-1, 37 nM	7.614	0.560	148	mTOR Inhibitor
	Torin-1, 12 nM	7.521	0.554	148	mTOR Inhibitor
	Clozapine, 30 μM	7.513	0.554	148	Antipsychotic Agent
1.1 μM	Torin-1, 12 nM	9.753	0.670	148	mTOR Inhibitor
	Torin-1, 37 nM	9.304	0.648	148	mTOR Inhibitor
	H1152, 3.3 μM	8.993	0.633	148	ROCK2 Inhibitor
3.3 μM	Albendazole (Albenza), 3.3 μM	8.363	0.601	148	Microtubule Inhibitor; Anthelmintic Agent
	Hydroxyfasudil, 90 μM	8.304	0.598	148	ROCK1, ROCK2 Inhibitor
	Fluvastatin, 10 μM	8.288	0.597	148	HMG-CoA Reductase Inhibitor
